# INITIATION OF INTERACTION AS THE BEGINNING OF SOCIAL PARTICIPATION: CONVERSATION ANALYSIS OF A JAPANESE SENIOR CLUB

**DOI:** 10.1093/geroni/igz038.617

**Published:** 2019-11-08

**Authors:** Eri Sakai, Akihiko Kamesawa, Riko Nakayama, Jihoon Kim, Akizuki Yuri, Yingxue Yang, Ryogo Ogino, Jun Goto

**Affiliations:** 1 Department of Sociology, Graduate School of Humanities and Sociology, The University of Tokyo, Tokyo, Japan; 2 The University of Tokyo, Bunkyo-ku, Tokyo, Japan; 3 Department of Integrated Education and Science, Graduate School of Education, The University of Tokyo, Tokyo, Japan; 4 the University of Tokyo, Tokyo, Japan; 5 Department of Urban Engineering, Graduate School of Engineering, The University of Tokyo, Tokyo, Japan; 6 Institute of Gerontology, Tokyo, Japan; 7 Institute of Gerontology, The University of Tokyo, Tokyo, Japan

## Abstract

The rate of social participation of senior citizens in a senior club’s activities is not equal to the rate of desire for the said participation. Earlier studies mainly examined personal and social factors which influence the participation rate, overlooking the practical methods by which senior citizens can overcome barriers to participating in club activities. Our study aims to clarify the features of a club activity as a resource by analyzing the activity’s interactions. Our study is based on data extracted from videotaped recordings of a senior calligraphy club in Kanto, Japan. In September 2018, one lecturer and 11 participants were videotaped for 3 hours, and the video underwent conversation analysis, which elucidates how people organize activities under specific circumstances. We analyzed how a female newcomer to the activity initiated face-to-face interaction, which is considered the first step of social participation. She talked to other participants who were familiar with the exercise several times by inquiring how to read kanjis on teaching materials. These findings suggest that visualization of skill relative to the other creates an environment for initiating face-to-face interaction. In this case, the newcomer utilized the difference in skill denoted by teaching materials and was given the rational reason to talk to the others already engaging in the activity. Therefore, designing teaching materials that assign the learning level of each participant may be effective in promoting social participation in senior study clubs.

